# Comparison of blogshots with plain language summaries of Cochrane systematic reviews: a qualitative study and randomized trial

**DOI:** 10.1186/s13063-020-04360-9

**Published:** 2020-05-25

**Authors:** Ivan Buljan, Ružica Tokalić, Marija Roguljić, Irena Zakarija-Grković, Davorka Vrdoljak, Petra Milić, Livia Puljak, Ana Marušić

**Affiliations:** 1 Department of Research in Biomedicine and Health, University of Split School of Medicine, Šoltanska 2, 21000 Split, Croatia; 2 Center for Evidence-based Medicine and Health Care, Catholic University of Croatia, Split, Croatia; 3 Department of Oral Diseases and Periodontology, University of Split School of Medicine, Split, Croatia; 4 Department of Family Medicine, University of Split School of Medicine, Split, Croatia; 5 University of Split School of Medicine, Split, Croatia

**Keywords:** Patient education, Medical decision making, Health communication, Evidence-based medicine

## Abstract

**Background:**

Cochrane, an organization dedicated to the production and dissemination of high-quality evidence on health, endeavors to reach consumers by developing appropriate summary formats of its systematic reviews. However, the optimal type of presentation of evidence to consumers is still unknown.

**Objective:**

The aim of this study was to investigate consumer preferences for different summary formats of Cochrane systematic reviews (CSRs), using both qualitative and quantitative approaches.

**Methods:**

Initially, we conducted three focus groups with medical students (*n* = 7), doctors (*n* = 4), and patients (*n* = 9) in 2017 to explore their health information search habits and preferences for CSR summary formats. Based on those findings, we conducted a randomized trial with medical students at the University of Split School of Medicine, Croatia, and with patients from three Dalmatian family practices to determine whether they prefer CSR blogshots (*n* = 115) or CSR plain language summaries (PLSs; *n* = 123).

**Results:**

Participants in the focus groups favored brief and explicit CSR summary formats with fewer numbers. Although we found no difference in participants’ preferences for a specific summary format in the overall sample, subgroup analysis showed that patients preferred blogshots over PLSs in comparison to medical students (*P* = 0.003, eta squared effect size η^2^ = 0.04).

**Conclusion:**

CSR summaries should be produced in a format that meets the expectations and needs of consumers. Use of blogshots as a summary format could enhance the dissemination of CSRs among patients.

**Trial registration:**

The trial was registered in ClinicalTrials.gov, NCT03542201. Registered on May 31st 2018.

## Background

Recommendations for presenting health information to lay consumers include short formats, framing the results in a positive direction, using plain language, and situating the results in context [1]. Several systematic reviews provide strong evidence that decision aids, like written information materials, help people increase their knowledge, which is crucial in decision-making [2, 3]. However, there is still little evidence about which type of information material is superior to the others in terms of change in knowledge, attitudes, and behavior [2]. Lay consumers are rarely included in the development of written materials targeting them [4], and there is still no evidence that any intervention improves health literacy [5], which would help the lay population make health decisions independently. For these reasons, identification of optimal formats of health information would be important for informed decision-making.

Studies that identify optimal formats for information presentation to lay consumers are also important for organizations that are involved in the production and dissemination of health information to the public. One such organization is Cochrane, an international organization globally respected as the producer of high-quality evidence about health interventions in the form of Cochrane systematic reviews (CSRs). Cochrane is going to great lengths to present this evidence to lay consumers in formats that are acceptable, easily accessible, and comprehensible [6]. These include plain language summaries (PLSs)—a brief summary of a systematic review written in plain language—and infographics—a visual presentation accompanied with simple text. Despite standards and style guidelines, Cochrane PLSs remain diverse, varying in size and structure [7].

Although infographics were previously considered more suitable for consumers, compared with standard textual summaries [8], we recently showed in a randomized trial that consumers’ preference for infographics over textual summaries is very small and that both formats lead to similar knowledge outcomes [9].

The aim of this study was to explore consumers’ preferences for different summary formats of CSRs. Based on our recent comparison of PLSs and infographics [9], we first conducted focus groups with different stakeholders to explore their preferences for the presentation of findings from CSRs and suggestions on how it could be improved. The findings from the focus groups guided us in the choice of formats to be tested as a health information tool in a randomized controlled trial. We decided to test blogshots—short textual information about a systematic review on a simple graphic template, easily shared on social media [10]—which Cochrane has recently started developing, as a new format for presenting information.

## Methods

### Qualitative study

#### Study design

We conducted three focus groups between April and December 2017. We used purposive sampling in order to combine three different types of stakeholders, all consumers involved in the use of health information: third-year medical students (*n* = 7), doctors working in hospitals (*n* = 4), and patients (*n* = 9). Focus groups were homogenous, and all participants were considered as consumers of Cochrane information. The participants received two infographics and two PLSs and answered the same pre-determined questions.

#### Setting, participants, and procedure

All patients were members of the “Parents in Action” (RODA) non-governmental organization, dedicated to pregnancy and breastfeeding (http://www.roda.hr/). The focus groups with medical students and doctors were held at the University of Split School of Medicine in Split, Croatia, and the focus group with patients was conducted at the RODA facilities in Zagreb, Croatia. The patients were invited via the Cochrane Croatia liaison for lay consumers to participate in a discussion about the comprehension of health information, and she was present at the focus group (IZG) with the principal researcher (IB). Medical students who were interested in Cochrane and had contributed to PLS translations into Croatian were invited verbally to participate by a representative of Cochrane Croatia outside of and unrelated to any of their courses. Doctors from the School of Medicine were invited using publicly available e-mail contacts. We approached practicing physicians who had experience with Cochrane work. Out of five doctors invited, one could not participate due to clinical obligations, and the final sample consisted of four doctors practicing different specialties: emergency medicine, dental medicine, respiratory medicine, and pathology. All invited participants signed an informed consent form before the start of the focus group. During the focus groups with students and doctors, only the lead researcher (IB, a male interviewer with a degree in psychology and previous experience in qualitative research) and the participants were present in the room. Each focus group lasted around an hour. Participants were presented with four summary formats in total: two infographics, one in Croatian and one in English; and two plain language summaries, one in Croatian and one in English. We did this because we wanted to determine whether information needs to be presented in native language to be understood. Summary topics were: “External cephalic version for breech presentation before term” [11] and “External cephalic version for breech presentation at term” [12], which were used in our previous trial [9] and which were familiar to the participants from the RODA group.

#### Data collection and analysis

The questions for the focus group were prepared in advance, and were identical for all three focus groups:

1. Where do you search for health information? (Doctors were asked about information search strategies both for themselves and their patients.)
2. How similar is that information compared to these you have just read?
3. How did you find the textual summary?
4. How did you find the infographic?
5. In your opinion, which type of presentation is better and why?
6. Which improvements would you suggest regarding the summaries?
7. In your opinion, what should be improved in the dissemination of health information?

Participants were also permitted to discuss issues among themselves, expand on the themes from the questions, and support their statements with examples.

Data analysis included the coding of transcripts, categorization of initial codes, and generation of themes. Focus group conversations were recorded with an audio recording device and transcribed to Microsoft Word by the principal investigator. The transcripts were coded using R for Qualitative Data Analysis (RQDA) [13]. The participants’ identities were anonymized by the lead investigator (IB) (coded as P1–P7 for the medical student group, P8–P12 for the physician group, and P13–P20 for the patient group). Two researchers (IB, RT) conducted the initial coding of themes based on the participants’ comments. Preliminary themes were derived during the analysis based on the focus group questions and then iteratively refined using constant comparative approach [14], also allowing the identification of new themes in addition to the existing ones. One researcher performed the initial coding (IB), which resulted in many codes, and then the second researcher (RT) revised the codes until reaching a consensus, and the same procedure was applied in the higher categorization of code, until both researchers reached a consensus. Each code was placed in a single theme category only and saturation was achieved if the code was present in all three focus groups. After the first two focus groups saturation was achieved, and as the third focus group did not add any new themes, we did not enroll any more participants, bringing the total sample to 20 participants. In order to confirm validity of the identified themes, the summaries of the themes identified during the focus groups were sent to the participants after the derivation of the themes. Quotes of participants’ statements were translated from Croatian into English by one of the authors (IB) and checked for accuracy by another (AM).

### Randomized trial

#### Study design

We conducted a randomized controlled trial (RCT) in which we used two types of evidence summaries: Cochrane blogshots and Cochrane PLSs. We chose CSRs that addressed common health issues of general interest to consumers, regardless of their medical knowledge: headache and fractures [15, 16].

The blogshots of the corresponding PLSs were taken from the Cochrane blogshot archive (https://cochraneblogshots.tumblr.com/). The blogshots had a maximum of three bullet points, including the title, main conclusion, number of studies, and number of participants. After the identification of suitable blogshots, we searched for the corresponding PLSs. Both PLSs were structured and had a similar amount of text. In order to standardize the language characteristics of the formats, all PLSs were first checked using the IBM Watson Tone Analyzer [17, 18] and then refined to ensure similarity in the emotional tone and sentiment, so that each summary had similar contents of three emotional tones: sadness, analytic, and tentativeness (total—over 50% for each of the tones). In the refinement process, we carefully revised the summaries so that the meaning and the message of the sentences remained the same. Summaries were also standardized for visual format: they were under 500 words long and consisted of four paragraphs with the following headings: “What is this (review) about?”, “Why is it important?”, “What did we find?”, and “What is the quality of the evidence?”

Both PLSs and blogshots were translated into Croatian and back-translated by a professional translator to ensure the validity of the translation. There were no significant changes after back-translation.

#### Setting and participants

We performed a randomized, parallel, two-arm double blind trial at the University of Split School of Medicine, in three family practices in Split and on the island of Brač, and at the University Hospital of Split. Participants at the School of Medicine were second- to fifth-year pharmacy students, first- and second-year dentistry students, and third-year medical students, who were recruited by a member of the research team, who invited them to fill in the questionnaires at the end of unrelated courses. The participants at the University Hospital and family practices were patients ≥ 18 years of age who were recruited by their doctor by inviting them to fill out a questionnaire in the waiting room. Data collection was performed from March to June, 2018.

#### Intervention

A Cochrane blogshot was the intervention and a PLS was the comparator. The intervention was delivered in the form of a paper and pen questionnaire. Each participant received a questionnaire with only one type of evidence summary format—blogshot or PLS. The questionnaire contained two summaries of the same format (either PLSs or blogshots) to control for different health topics. Each summary was followed by a set of questions about its content.

The questionnaire was organized so that the title page was the same for all surveys, regardless of the type of summary format. Researchers who delivered the survey to the participants could not see which format they were distributing because both groups had the same title page. The evidence summary format was always presented on the left page of the survey, while the questions and answers were on the right side of the survey, to make it easier for participants to consult the summary format while answering the questions.

The translated surveys can be found in Additional file 1.

#### Outcomes

##### Primary outcome

The primary outcome was participants’ preference for presentation format. After reading the summary format, each participant reported on whether the presented format is a suitable way of presenting health information, on a scale from 1 (“do not agree” at all) to 10 (“fully agree”); the total score for both formats in each questionnaire ranged from 2 to 20.

##### Secondary outcomes

The secondary outcomes were:

1. Efficacy of the described treatment for the described medical condition on a scale from 1 (“do not agree” at all) to 10 (“fully agree”); the total score for both formats in each questionnaire ranged from 2 to 20.
2. Comprehension of the content of the summary format. Comprehension was assessed using a brief knowledge test with four multiple choice questions for each of the two PLSs or two blogshots (one correct answer out of three offered; total possible test score ranged from 0 to 8).

##### Demographic data

On the title page of the survey, participants answered questions about their gender, age (in years), and education level. They also reported on the sources of health information they use (Internet, family and friends, books, family physician, something else) and preference for online health information sources (first page on Google, forums, hospital websites, national specialized websites, international specialized websites, scientific articles, and/or writing email to physicians). After reading the summary, the participants answered questions about their preference for this type of presentation format, content of the summary (comprehension), and perceived efficacy of the described treatment. For each summary format, the participants were also asked their preference towards numbers (subjective numeracy) in health communication. The final score on subjective health numeracy for each participant was expressed as an average of scores for each summary. On the last page of the survey, the participants were asked to take a five-item objective numeracy test with multiple choice questions [19], with a total score ranging from 0 (all questions wrong) to 5 (all questions correct).

#### Randomization

**Fig. 1:**
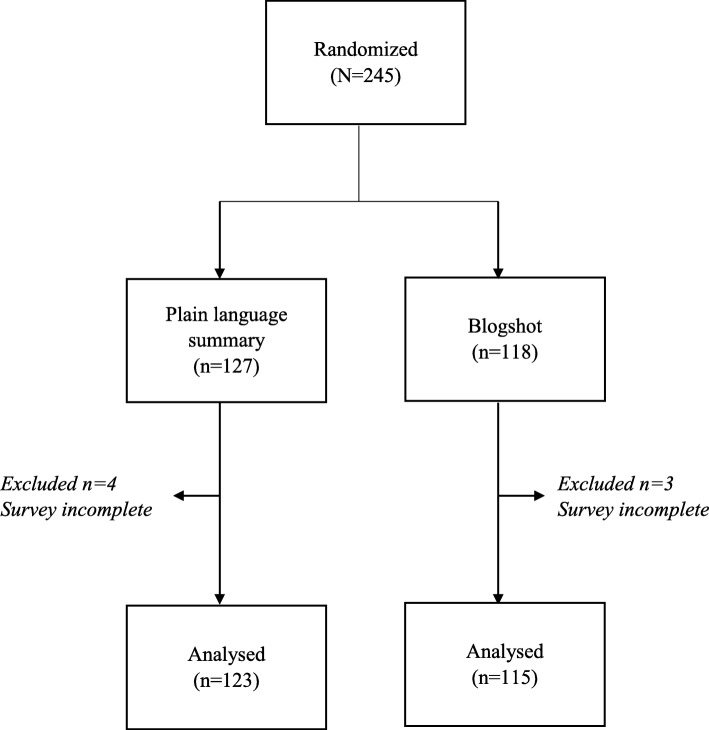
Flowchart of participants in study comparing Cochrane plain language summaries and Cochrane blogshots

Randomization was conducted using online software Research Randomizer (https://www.randomizer.org/), using the block randomization approach. Intervention questionnaires were then arranged according to the randomization order (Fig. 1).

#### Blinding

Researchers involved in randomization were not involved in the allocation of participants because researchers who sorted the surveys in random order differed from those who distributed the surveys to the participants. The surveys had the same first page, regardless of the trial group. Each distributor of the intervention questionnaires was given a package of randomly sorted questionnaires, with the first page facing up in an envelope, and instructed to distribute them to the participants in order from the top of the package.

The participants were blinded to study design and randomization. They were asked to participate in a survey about the presentation of health information and monitored while they took the survey. Students completed the surveys before the start of their lectures, monitored by their course teacher, while patients took the survey in the waiting room, while waiting for their doctor’s appointment, and were monitored by a nurse.

#### Sample size

We calculated the sample size based on data from previous research for the overall population to detect a difference in average scores on preference for a specific format (M_diff_ = 12.3, SD_1_ = 9.6, SD_2_ = 8.5) between different summary groups (CSR PLSs and scientific abstract) [9]. We calculated that we would need ten participants in each group to obtain the desired difference (20 in total), for 0.05 level alpha, and 80% of the power. For the sample size calculation, we used MedCalc v.17.9.4. (MedCalc Software bvba, Ostend, Belgium, 2017).

#### Data analysis

All data collected were anonymous and stored on a secure server at the University of Split School of Medicine. All statistical analyses were performed using JASP v.0.9.0.0 (JASP Team, 2018). Participants who did not complete the survey were excluded from analysis. Gender, level of education, sources of health information, and Internet sources were presented as frequencies and percentages. Numeracy scores were presented as median values with interquartile range.

Preference for health information presentation, perceived efficacy of the treatment, and comprehension scores were presented as means with 95% confidence intervals. The differences between formats (PLS and blogshot) were initially tested on the entire sample using the *t*-test and mean differences. In subgroup analyses, the differences between the formats (PLS and blogshot) and groups (medical students vs patients) were tested using two-way ANOVA (2 × 2 factorial design), in order to avoid alpha error. Effect sizes were expressed using eta squared (η^2^).

## Results

### Qualitative study

In the focus group analysis, five common themes emerged. Quotes from the focus group participants are available in Appendix B. The findings describe the practices of seeking health information online, issues in comprehension of evidence, and advice on how to improve information search and translation. The final sample consisted of seven medical students (five women, age range 21–22 years), nine patients (all women, age range 30–42 years, all with a university education), and four doctors (two women, age range 29–35 years).

#### Influence on the choice of health information source

Participants elaborated that there were various factors which influenced their choice of health information sources, but that the main factor was trust in the source. Therefore, each information source, in order to successfully present the information to the user, must have the trust of the user, while different users varied in their amount of trust for different sources. Participants emphasized that the average user/person looks for concise information. Also, according to participants, searching for health information depends on two factors: the type and seriousness of the health problem and the amount of available time. In general, their assumption was that the more time a person had and the more serious the health problem, the more time would be dedicated to searching for answers and more information sources would be used.

#### The Internet as the primary source of health information and other sources of information

Participants stated that the Internet was often the primary source of health information; other sources included books, friends, family, and doctors. Scientific websites were rarely visited. In Internet browsing, users often read only the first page of search results and/or read forums because they were interested in other patients’ experiences. Consequently, information searching online resulted in very narrow and rarely scientifically supported content. The main reason for such a practice was participants’ lack of awareness of Cochrane’s website, except for a few individuals, mostly those well-educated. Also, the Cochrane website was perceived as not adjusted to a wide range of users.

#### Issues in comprehension of current scientific formats

The main issues in comprehension for lay health consumers were the presentation of numbers and understanding of uncertainty. Participants perceived that people have difficulties with the presentation of numbers regardless of the format of presentation, and the recommendations were that the amount of numeric data should be decreased to a minimum or eliminated, and that they should be presented both visually and textually. Also, the patients wanted to find clear cut answers to health questions, which science can rarely provide, and they did not understand the concept of study quality. According to participants’ statements, patients stop searching for health information when they find a concrete answer, even if it is not correct, and do not read texts with scientific answers because they contain too many numbers and do not provide an explicit answer.

#### Doctors and patients have communication issues

Participants thought that doctors have issues with tracking health information and evidence because science is constantly changing; doctors are afraid of risks of new therapies, so they rather stick to the old ones; and very few of them use Cochrane evidence. Patients felt that communication between themselves and doctors is unsatisfactory and that doctors do not want to listen to their views, even when they are supported by evidence. On the other hand, doctors reported that they have too many patients on a daily basis and are therefore unable to give them enough attention in order to present them with more treatment options, advice on changing health habits, and/or explanations about evidence in health.

#### Recommendations for improvement

Participants stated that health information should be easily available, structured, consistent in presentation, explicit, brief, using plain language, and with numbers presented in a table and/or visually. Textual information was considered enough; if visual information is needed, it should be limited to a single table and/or image.

### Rationale for the choice of blogshot format for the RCT

After the focus groups, we assessed the suitability of various summary formats produced by Cochrane that would match the requirements from the focus groups: being short, easily available, explicit, using plain language, and with only a few or no numbers. CSR scientific abstracts use complex language, usually with many numbers. Press releases vary in size and structure, language used can be complex, and there are only a very limited number of press releases. Infographics were not preferred by the participants of the focus groups as they perceived that format as difficult to present and design. Hence, we decided to test blogshots as a novel format produced by Cochrane, given that they are brief, consistent in presentation, written in plain language, and can be easily shared.

### Randomized trial

#### Sample characteristics

Most of the participants in the randomized trial were women, with at least a high school education (Table 1). The family doctor and the Internet were the most prevalent sources of health information and a very low proportion of participants reported that they read scientific articles as a source of health information (Table 1).

**Table 1:** Sample characteristics in a randomized trial comparing blogshots with plain language summaries (*n* = 238)

**Trial**	**Presentation format**			
**Variables**	**Blogshot (*n* = 115)**		**PLS (*n*= 123)**	
	**Medical students (*n*** **= 37)**	**Patients** **(*n*** **= 78)**	**Medical students (*n*** **= 41)**	**Patients (*n*** **= 82)**
**Women (%)**	29 (78.4)	52 (66.7)^a^	33 (80.5)^a^	48 (58.4)^a^
**Age (Md, IQR)**	21 (20 to 22)	46 (32 to 62)	21 (21 to 22)	51 (37 to 65)
**Education (%)**				
Elementary	0	4 (5.1)	0	3 (3.7)
High school	0	36 (46.2)	0	40 (48.8)
Currently enrolled in university	37 (100.0)	5 (6.4)	41 (100)	7 (8.5)
College graduate	0	11 (14.1)	0	11 (13.4)
University graduate	0	22 (28.2)	0	20 (24.4)
PhD	0	0	0	1 (1.2)
**Information sources (%)**^b^				
Internet	31 (83.8)	49 (62.8)	35 (85.4)	52 (63.4)
Family and friends	8 (21.6)	29 (37.2)	10 (24.4)	30 (36.6)
Books	23 (62.2)	16 (20.5)	25 (60.9)	16 (19.5)
Family doctor	20 (54.1)	65 (83.3)	15 (36.6)	60 (73.2)
**Internet sources (%)**^b^				
First page provided by Internet search engine	9 (24.3)	32 (41.0)	17 (41.5)	27 (33.0)
Forums	9 (24.3)	26 (33.3)	13 (31.7)	26 (31.7)
Hospital websites	10 (27.0)	10 (12.8)	7 (17.1)	12 (14.6)
Local specialized websites	19 (51.4)	17 (21.8)	22 (53.7)	25 (30.5)
International specialized articles	5 (13.5)	13 (16.7)	4 (9.8)	9 (10.9)
Scientific articles	11 (29.7)	7 (8.9)	11 (26.8)	9 (11.0)
Email to physicians on Internet websites	1 (2.7)	2 (2.6)	2 (4.9)	1 (1.2)
**Numeracy preference item**^c^**(Md, IQR)**	1 (−2 to 2)	1 (−1 to 2)	1 (0 to 2)	0 (−2 to 3)
**Objective numeracy (Md, IQR)**^d^	5 (4 to 5)	3 (3 to 4)	4 (4 to 5)	3 (2 to 4)

*Md* median, *IQR* interquartile range

^a^ One answer missing

^b^ Multiple entries allowed

^c^ One item ranging from − 4 (indicating absolute preference towards words in presentation of health information) to + 4 (indicating absolute preference towards numbers in presentation of health information)

^d^The scale was calculated as the sum of correct answers, range from 0 to 5

#### Comparison of blogshots and PLSs

In the overall sample, no difference was found in perceived efficacy of the described treatment or preference for certain format between participants who read the blogshots or PLSs (Table 2). Participants who read blogshots answered more questions correctly about the content of the CSR compared to those who read PLSs, with small effect size (Table 2).

**Table 2:** Comparison of Cochrane blogshots and PLSs regarding preference for presentation type perceived efficacy of described treatment and comprehension scores (*n* = 238)

**Variable**^**a**^	**Mean, 95% CI**		**Mean difference(95% CI)**	***P***^**b**^
	**Blogshot (*n* = 115)**	**PLS (*n* = 123)**		
Preference for health information presentation (score 2–20)	12.25 (11.10 to 13.24)	12.32 (11.34 to 13.33)	−0.03 (− 1.41 to 1.34)	0.963
Perceived efficacy of described treatment (score 2–20)	12.81 (11.91 to 13.72)	12.23 (11.22 to 13.19)	0.58 (−0.76 to 1.92)	0.395
Comprehension (score 0–8)	7.01 (6.64 to 7.38)	6.46 (6.08 to 6.85)	0.55 (0.02 to 1.074)	0.043

*CI* confidence interval, *PLS* plain language summary

^a^ The total score on perceived efficacy and preference for health information is calculated as the sum of scores for both summaries in respective groups. Comprehension is calculated as the sum of correct answers for two summaries

^b^*t*-Test for independent samples

Subgroup analysis revealed an interaction effect in that medical students preferred PLSs and had higher perceived efficacy of the drug when presented in a PLS format, whereas patients preferred blogshots and gave higher scores on perceived efficacy when presented with a blogshot compared to a PLS (Table 3). Compared to patients, medical students had more correct answers about the content of the summary, regardless of the summary format (Table 3).

**Table 3:** Comparison of blogshots and PLSs between patients and medical students regarding preference for presentation type, perceived efficacy of the described treatment and comprehension scores (*N*=238) (*N*=238)

	**Group (mean, 95% CI)**						
	**Patients**		**Medical students**				
**Variable**	**Blogshot (*n*=78)**	**PLS (*n*=82)**	**Blogshot (*n*=37)**	**PLS (*n*=41)**	**P(η**^**2**^**)**^**a**^	**P (η**^**2**^**)**^**b**^	**P(η**^**2**^**)**^**c**^
Preference for health information presentation (score 2-20)	13.37 (12.16 to 14.53)	11.98 (10.82 to 13.13)	9.95 (8.23 to 11.66)	12.90 (11.27 to 14.53)	0.280	0.093	0.003 (0.04)
Perceived efficacy of described treatment (score 2-20)	13.59 (12.43 to 14.75)	11.83 (10.70 to 12.96)	11.16 (9.48 to 12.85)	13.02 (11.43 to 14.62)	0.944	0.392	0.012 (0.03)
Comprehension (score 0-8)	6.68 (6.23 to 7.13)	6.02 (5.59 to 6.40)	7.70 (7.05 to 7.92)	7.34 (6.73 to 7.96)	0.068	<0.001 (0.07)	0.596

*CI* confidence interval, *PLS* plain language summary, *η*^*2*^ eta squared

^a^ Main effect: format, Two-way ANOVA, df=1/215

^b^ Main effect: sample (patients vs medical students)

^c^ Interaction of main effects of format and sample

## Discussion

Based on the issues emerging from the focus groups, we compared blogshots, as a very brief and explicit summary format, to PLS, as a standard textual summary for lay consumers. The trial group reading blogshots did not differ overall in the format preference from the group reading PLSs, but they had significantly higher comprehension scores. Patients preferred blogshot presentation over PLSs, whereas medical students showed greater preference towards PLSs compared to blogshots.

Previous research has indicated that obtaining health information online can potentially lead to undesirable outcomes [20]. Lay consumers are generally unaware of sources where they may find scientifically supported information [21], and they expect their doctors to keep up with new scientific discoveries. However, doctors in our qualitative study admitted that it was hard for them to keep up with new information because of the large number of patients they see. Besides the lack of time, there is some evidence that interventions for search of evidence-based health information are not effective [22].

We found no difference in preference between blogshot and PLS format in regard to presentation or perceived efficacy of the described treatment, but participants in the blogshot group had significantly higher comprehension scores. A possible reason could be that PLSs contain a lot of information, which patients may find irrelevant for their question. On the other hand, blogshots are a simple, concise format easily adaptable to different devices (website, app, and other online touchpoints) and therefore could be a suitable format to engage with the widest community.

Medical students scored significantly higher on comprehension compared to patients. Previous findings suggest that it is hard even for experienced doctors to understand treatment effects, and that they best understand dichotomous outcomes [23]. On top of that, medical students assessed a treatment as more effective when it was presented as a PLS, while patients gave higher assessment of the efficacy of a treatment when it was presented as a blogshot. A possible reason why medical students gave higher scores for intervention efficacy when review methodology was described, compared to patients, is that medical students were more aware of the modest effects of treatments in practice, while patients would expect an effective treatment to make a very significant difference.

In our study, both in its qualitative and quantitative part, participants reported that they rarely specifically searched for evidence-supported information, like scientific articles, or international or local specialized websites. For most of them, browsing for health information was mostly limited to the first hits retrieved by Internet search engines or to forums, where they could read about other people’s experiences. The conclusion to be drawn from this finding is that lay consumers are still unfamiliar with the concepts of evidence-based medicine and its use in everyday health decision making. It is not known how much the initiatives like *Testing Treatments* for promoting critical thinking about treatment claims, which provide information in 14 languages [24], are familiar to the public in general and specifically in Croatia, where the Croatian edition of *Testing Treatments* is also freely available online. National educational campaigns and education from an early age may be the solution to this problem, as there is evidence that interventions to improve critical assessment in health are effective even in school children [25].

One of the strengths of this study was the inclusion of different stakeholders in the comparison of different formats of evidence summaries. The focus group discussion enabled us to explore stakeholders’ preferences and issues in comprehension of evidence, and to draw conclusions about optimal format type, which is the newly proposed way for designing randomized controlled trials in testing health interventions [26]. To our best knowledge, this is the first study to compare consumers’ preference and comprehension of Cochrane blogshots and other types of evidence summaries, including diverse populations: patients and medical students.

Our findings should be interpreted in view of several limitations. We conducted three focus groups, with 20 participants in total. However, despite the small number of participants, after the two initial focus groups no new information emerged in the third focus group, so that saturation of themes was achieved. Although focus group participants were presented with summary formats for two CSRs, they served only as a starting point for discussion about the optimal type of information; providing more summaries could possibly cause fatigue and confusion among participants. In the randomized trial, we did not have information on how many patients refused to participate because the trials were performed in distant family practices. Therefore, the interpretation of results must take into consideration that patients who were motivated and those who had potentially greater knowledge about health evidence may have volunteered for the trial. Such patients may have higher levels of health literacy and health numeracy, as shown in other studies [27]. Although we sorted the surveys in a randomized order in specially prepared packages, and gave specific instructions for their distribution, we cannot guarantee that the distributors (nurses or doctors in family medicine offices and teachers at the medical school) respected these instructions and remained blinded. In balancing the bias from the possibility of unblinding and the bias from having the creators of the questionnaire and the trial deliver the intervention, we decided that the former bias was smaller. The study was also limited in the development of questions for the two formats, as the answers offered in relation to the comprehension of information and efficacy of the treatment had to be present in both the PLS and blogshot format. In the randomized trial, the participants in each arm were presented with a single format, so they did not have a reference point. We did not find differences for our primary outcome regarding format preference in the overall sample, but we did find differences in comprehension and, although those differences were small, this finding needs to be further explored in future research. In the assessment of their reasoning abilities, we used the five-item numeracy test, which is a very concise measure of health numeracy [19]. We did not use a health literacy test, which addresses a broader concept than health numeracy but is more subjective and culturally related. Also, a higher proportion of women in our sample reflects the gender structure of the University of Split School of Medicine, where the majority of students are female (e.g. [9, 28] and in the patient population the distribution reflects the findings of other studies which report that women are more willing to participate in health research surveys [29]. However, we do not think that the higher proportion of women could significantly affect the overall results, but future research should bear in mind that samples should be gender balanced. Finally, the study was performed in a narrow geographical setting (Croatia), so trials in other settings are needed to confirm the generalizability of our findings.

## Conclusion

While no differences were found between groups regarding preference for blogshots or PLSs, the participants in the blogshot group had higher scores on comprehension. Subgroup analysis showed that patients had greater preference for blogshots, whereas students showed greater preference towards PLSs. Future research should explore the use of blogshots in raising awareness and dissemination of Cochrane evidence.

## Supplementary information

**Additional file 1.** Randomized controlled trial – materials used as intervention; Qualitative study – participant quotes.

## Data Availability

The datasets analysed during the current study are available from the corresponding author on reasonable request.

## Abbreviations

PLS: Plain language summary
CSR: Cochrane systematic review
RQDA: R for qualitative data analysis
