# Supplementary material for: Comparison of blogshots with plain language summaries of Cochrane systematic reviews: a qualitative study and randomized trial
Source: Trials. 2020 May 25;21:426. doi: 10.1186/s13063-020-04360-9 (PMC7249676; doi:10.1186/s13063-020-04360-9)
Supplement: Supplementary file 1 — Additional file 1. Randomized controlled trial – materials used as intervention; Qualitative study – participant quotes. [file 13063_2020_4360_MOESM1_ESM.docx]

**Additional file 1**

**Randomized controlled trial, materials used in research**

**a) PLS format**


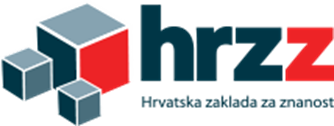

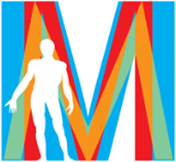


Projekt ProHealth

**Sveučilište u Splitu**

**Medicinski fakultet**

**Universitas Studiorum**

**Spalatensis**

**Facultas Medica**

**Questionnaire about health information**

Dear participant, the questionnaire before you is a part of the research project “Professionalism in Health: ProHealth” financed by Croatian Science Foundation. In this research we want to determine the optimal format of health information presentation. Your responses will be fully anonymized (gender and age data cannot reveal your identity) and will be used for research purposes only. In this questionnaire, your task will be to read brief descriptions of scientific research and answer questions about them. Continuing with this survey you give your consent to participate in the study.

**Demographic data:**

**Gender M F**

**Age**: _____________________ (In years)

**Education degree:**

a) Elementary school degree

b) High school degree

c) Currently enrolled in college/university

d) College degree

e) University degree

f) PhD degree

**Which sources do you rely on when you search for health information (Choose everything that applies to you):**

a) Internet

b) Family and friends

c) Books

d) Family doctor

e) Something else (please describe): ______________________________________-

**If you have chosen Internet as the source of health information, please answer the following question:** Which sources on the Internet do you rely on when you search for health information (Choose everything that applies to you):

a) I usually read anything that pops out first on an Internet search engine after I enter the terms of interest

b) I read Internet forums to obtain the information I search for

c) I browse he hospital websites or websites of specialized health institutions

d) I browse domestic health related websites (e.g. PlivaMed)

e) I browse international health related websites (e.g. Cochrane.org)

f) I search for research articles in scientific databases

g) I write an email to physicians available on Internet portals (e.g. Where is the evidence, cybermed.hr)

**Oral paracetamol for treatment of acute episodic tension-type headache in adults**

**What is this about?**

People with frequent episodic tension-type headache have between two and 14 headaches every month. Tension-type headache limits people concentration and working properly, and may result in much disability. When headaches do occur, they get better over time, even without treatment. Paracetamol is an often used painkiller, available without prescription (over the counter) in most parts of the world. The usual dose is 1000 mg (usually two tablets) taken by mouth.

**What has been done?**

In October 2015, we searched the medical literature and found 23 studies involving 8079 participants looking at paracetamol for frequent episodic tension-type headache.

**What evidence did we find?**

About 6000 participants were involved in comparisons between paracetamol 1000 mg and placebo (a dummy tablet). Results were usually reported two hours after taking the medicine or placebo. The outcome of being pain free at two hours was reported by 2 in 10 people taking paracetamol 1000 mg, and in 1 out of 10 people taking placebo, meaning that only 1 in 10 people benefited because of paracetamol (high quality evidence).

There was no difference between paracetamol and placebo in occurrence of side effects (high quality evidence). Most side effects were mild or moderate in intensity. No side effects were serious.

We found a very small amount of information comparing paracetamol 500 mg to 650 mg with placebo, and comparing paracetamol 1000 mg with other painkillers. There was no difference between any of these treatments.

**How good is the evidence?**

The quality of the evidence was moderate or high for paracetamol 1000 mg compared with placebo, and low or very low for paracetamol 500 mg to 650 mg compared with placebo, and for paracetamol 1000 mg compared with other painkillers. High quality evidence means that we are very certain about the results. Low quality evidence means that we may not be certain about the results.

**Please answer the questions about the text**

1. How often did the participants have headaches in the report you read?

a) 2-14 times a month b) 2-14 times a year c) It is not mentioned

2. How much time minimum had to pass to relieve the pain to prove the paracetamol effectiveness?

a) it depends how serious the pain was b) one hour c) two hours

3. With what the paracetamol was compared in the report you read?

a) with another drug b) with fake drug c) with nothing

4. How often did the participants have to take the paracetamol?

a) every day b) after two hours c) when they suffered from headache

**In the following three questions there are no correct or incorrect answers. We only ask your opinion about the present statements. Please choose the number which describes your opinion the most.**

**5. I consider that the paracetamol is effective in tension headaches treatment.**

| **I do not agree at all** |  |  |  |  |  |  |  |  | **I agree completely** |
| --- | --- | --- | --- | --- | --- | --- | --- | --- | --- |
| 1 | 2 | 3 | 4 | 5 | 6 | 7 | 8 | 9 | 10 |

**6. This is the appropriate format for health information presentation.**

| **I do not agree at all** |  |  |  |  |  |  |  |  | **I agree completely** |
| --- | --- | --- | --- | --- | --- | --- | --- | --- | --- |
| 1 | 2 | 3 | 4 | 5 | 6 | 7 | 8 | 9 | 10 |

**7. Please asses which way of presentation of health information do you prefer (-4 means that you prefer only words, and +4 means that you prefer only numbers)**

| **I prefer only words** |  |  |  |  |  |  |  | **I prefer only numbers** |
| --- | --- | --- | --- | --- | --- | --- | --- | --- |
| -4 | -3 | -2 | -1 | 0 | +1 | +2 | +3 | +4 |

**8. If you think that you would need another information to make a decision about the effectiveness of the paracetamol, what would it be? Please state your opinion**

__________________________________________________________________________.

**Vitamin D and related vitamin D compounds for preventing fractures resulting from osteoporosis in older people**

**What is this about?**

Fractures are very common in post-menopausal women and older men due to age-related weakening of their bones (osteoporosis) and lead to serious disability or even death. Those who survive have problems with mobility and require greater social and nursing care.

Vitamin D is necessary for building strong bone and older people usually have low vitamin D levels due to lack of exposure to sunlight and low consumption of vitamin D in their diet. It has been suggested that taking additional vitamin D in the form of supplements helps to reduce the risk of fractures of the hip and other bones.

**What has been done?**

To investigate the effects of vitamin D or vitamin D-related supplements, taken with or without calcium supplements, for preventing fractures in post-menopausal women and older men, the review authors searched the medical literature up to December 2012, and identified 53 relevant medical trials, with a total of 91,791 participants.

**What evidence did we find?**

The trials reported fracture outcomes in postmenopausal women or men aged over 65 years from community, hospital and nursing-home settings. The trials examined whether the Vitamin D and related supplements are better at preventing fractures in comparison to fake supplements, no supplements or calcium alone. In some trials calcium was given with Vitamin D. The review found reliable evidence that taking vitamin D only will not prevent fractures. However, reliable evidence showed that vitamin D taken with additional calcium supplements slightly reduced the number of hip fractures and other types of fracture. The review found that there was no increased risk of death from taking vitamin D and calcium.

**How good is the evidence?**

Although the risk of harmful effects (such as gastrointestinal (stomach) symptoms and kidney disease) from taking vitamin D and calcium is small, some people, particularly with kidney stones, kidney disease, high blood calcium levels, gastrointestinal disease or who are at risk of heart disease should seek medical advice before taking these supplements.

**Please answer the questions about the text**

1. For which medical condition the effectiveness of vitamin D was tested?

a) kidney disease b) indigestion c) fractures

2. Which compound, along with vitamin D, can help patients who suffer from described medical condition?

a) Placebo b) calcium c) It is not mentioned

3. How many trials were included in this review?

a) 91791 b) one c) 53

4. How many people with this health medical condition was included in this review?

a) 91791 b) one c) 53

**In the following three questions there are no correct or incorrect answers. We only ask your opinion about the present statements. Please choose the number which describes your opinion the most.**

**5. I consider vitamin D with calcium compounds is effective in prevention of fractures which are caused by osteoporosis in older people.**

| **I do not agree at all** |  |  |  |  |  |  |  |  | **I agree completely** |
| --- | --- | --- | --- | --- | --- | --- | --- | --- | --- |
| 1 | 2 | 3 | 4 | 5 | 6 | 7 | 8 | 9 | 10 |

**6. This is the appropriate format for health information presentation.**

| **I do not agree at all** |  |  |  |  |  |  |  |  | **I agree completely** |
| --- | --- | --- | --- | --- | --- | --- | --- | --- | --- |
| 1 | 2 | 3 | 4 | 5 | 6 | 7 | 8 | 9 | 10 |

**7. Please asses which way of presentation of health information do you prefer (-4 means that you prefer only words, and +4 means that you prefer only numbers)**

| **I prefer only words** |  |  |  |  |  |  |  | **I prefer only numbers** |
| --- | --- | --- | --- | --- | --- | --- | --- | --- |
| -4 | -3 | -2 | -1 | 0 | +1 | +2 | +3 | +4 |

**8. If you think that you would need another information to make a decision about the effectiveness of the vitamin D and its compounds, what would it be? Please state your opinion**

__________________________________________________________________________.

**In the following five questions there is only one correct answer. Please choose only one answer you think it is correct.**

1. A medical study will randomly assign people so they are equally likely to get medicine A or medicine B. If there are 300 people in the study, about how many are expected to get medicine A?

a. 100 people

b. 150 people

c. 200 people

d. 250 people

2. Older age and smoking both increase the risk of a heart attack over time. David is now 50 years old and smokes. His risk of a heart attack in the next 10 years is 10%. If he continues to smoke which of the following could be his risk of a heart attack over the next 20 years?

a. 5%

b. 10%

c. 30%

d. 100%

3. James starts a new blood pressure medicine. The chance of a serious side effect is 0.5%. If 1000 people take this medicine, about how many would be expected to have a serious side effect?

a. 1 person

b. 5 people

c. 50 people

d. 500 people

4. The PSA (Prostate Specific Antigen) is a blood test that can be used to screen for prostate cancer. However, 30% of men who have an abnormal test result will turn out not to have cancer. John has an abnormal test result. What is the chance that John has prostate cancer?

a. 0%

b. 30%

c. 70%

d. 100%

5. Rebecca is treated for stage 2 breast cancer. The chance that the cancer will come back is 10% over 10 years. If Rebecca takes a new medicine, this chance will decrease by 30%. If 100 women like Rebecca take this medicine, how many are now expected to have breast cancer come back within 10 years?

a. 3 out of 100 women

b. 7 out of 100 women

c. 10 out of 100 women

d. 30 out of 100 women

THE END!

Thank you for your participation!

**b) Blogshot format**


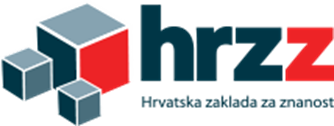

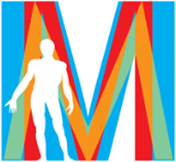


Projekt ProHealth

**Sveučilište u Splitu**

**Medicinski fakultet**

**Universitas Studiorum**

**Spalatensis**

**Facultas Medica**

**Questionnaire about health information**

Dear participant, the questionnaire before you is a part of the research project “Professionalism in Health: ProHealth” financed by Croatian Science Foundation. In this research we want to determine the optimal format of health information presentation. Your responses will be fully anonymized (gender and age data cannot reveal your identity) and will be used for research purposes only. In this questionnaire, your task will be to read brief descriptions of scientific research and answer questions about them. Continuing with this survey you give your consent to participate in the study.

**Demographic data:**

**Gender M F**

**Age**: _____________________ (In years)

**Education degree:**

a) Elementary school degree

b) High school degree

c) Currently enrolled in college/university

d) College degree

e) University degree

f) PhD degree

**Which sources do you rely on when you search for health information (Choose everything that applies to you):**

a) Internet

b) Family and friends

c) Books

d) Family doctor

e) Something else (please describe): ______________________________________-

**If you have chosen Internet as the source of health information, please answer the following question:** Which sources on the Internet do you rely on when you search for health information (Choose everything that applies to you):

a) I usually read anything that pops out first on an Internet search engine after I enter the terms of interest

b) I read Internet forums to obtain the information I search for

c) I browse he hospital websites or websites of specialized health institutions

d) I browse domestic health related websites (e.g. PlivaMed)

e) I browse international health related websites (e.g. Cochrane.org)

f) I search for research articles in scientific databases

g) I write an email to physicians available on Internet portals (e.g. Where is the evidence, cybermed.hr)

**Oral paracetamol for treatment of acute episodic tension-type headache in adults**


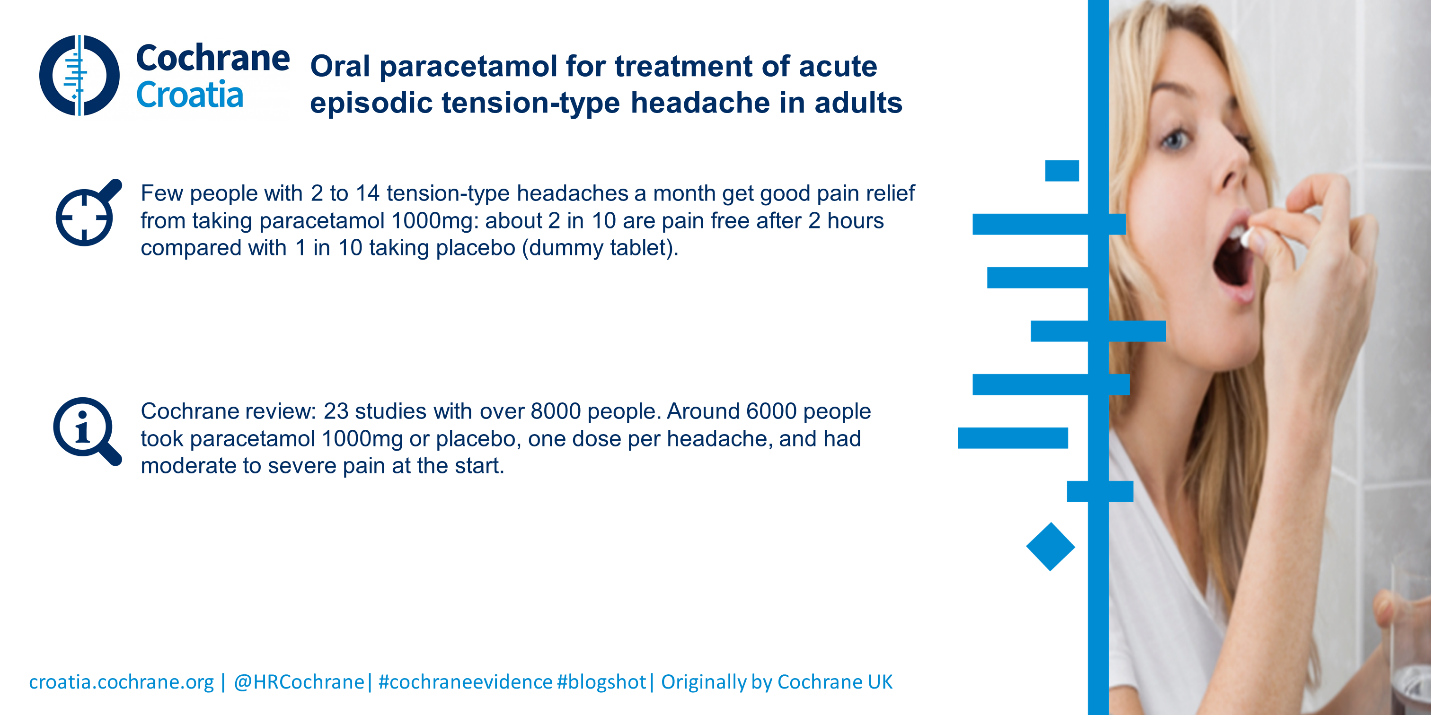


**Please answer the questions about the text**

1. How often did the participants have headaches in the report you read?

a) 2-14 times a month b) 2-14 times a year c) It is not mentioned

2. How much time minimum had to pass to relieve the pain to prove the paracetamol effectiveness?

a) it depends how serious the pain was b) one hour c) two hours

3. With what the paracetamol was compared in the report you read?

a) with another drug b) with fake drug c) with nothing

4. How often did the participants have to take the paracetamol?

a) every day b) after two hours c) when they suffered from headache

**In the following three questions there are no correct or incorrect answers. We only ask your opinion about the present statements. Please choose the number which describes your opinion the most.**

**5. I consider that the paracetamol is effective in tension headaches treatment.**

| **I do not agree at all** |  |  |  |  |  |  |  |  | **I agree completely** |
| --- | --- | --- | --- | --- | --- | --- | --- | --- | --- |
| 1 | 2 | 3 | 4 | 5 | 6 | 7 | 8 | 9 | 10 |

**6. This is the appropriate format for health information presentation.**

| **I do not agree at all** |  |  |  |  |  |  |  |  | **I agree completely** |
| --- | --- | --- | --- | --- | --- | --- | --- | --- | --- |
| 1 | 2 | 3 | 4 | 5 | 6 | 7 | 8 | 9 | 10 |

**7. Please asses which way of presentation of health information do you prefer (-4 means that you prefer only words, and +4 means that you prefer only numbers)**

| **I prefer only words** |  |  |  |  |  |  |  | **I prefer only numbers** |
| --- | --- | --- | --- | --- | --- | --- | --- | --- |
| -4 | -3 | -2 | -1 | 0 | +1 | +2 | +3 | +4 |

**8. If you think that you would need another information to make a decision about the effectiveness of the paracetamol, what would it be? Please state your opinion**

__________________________________________________________________________.

**Vitamin D and related vitamin D compounds for preventing fractures resulting from osteoporosis in older people**


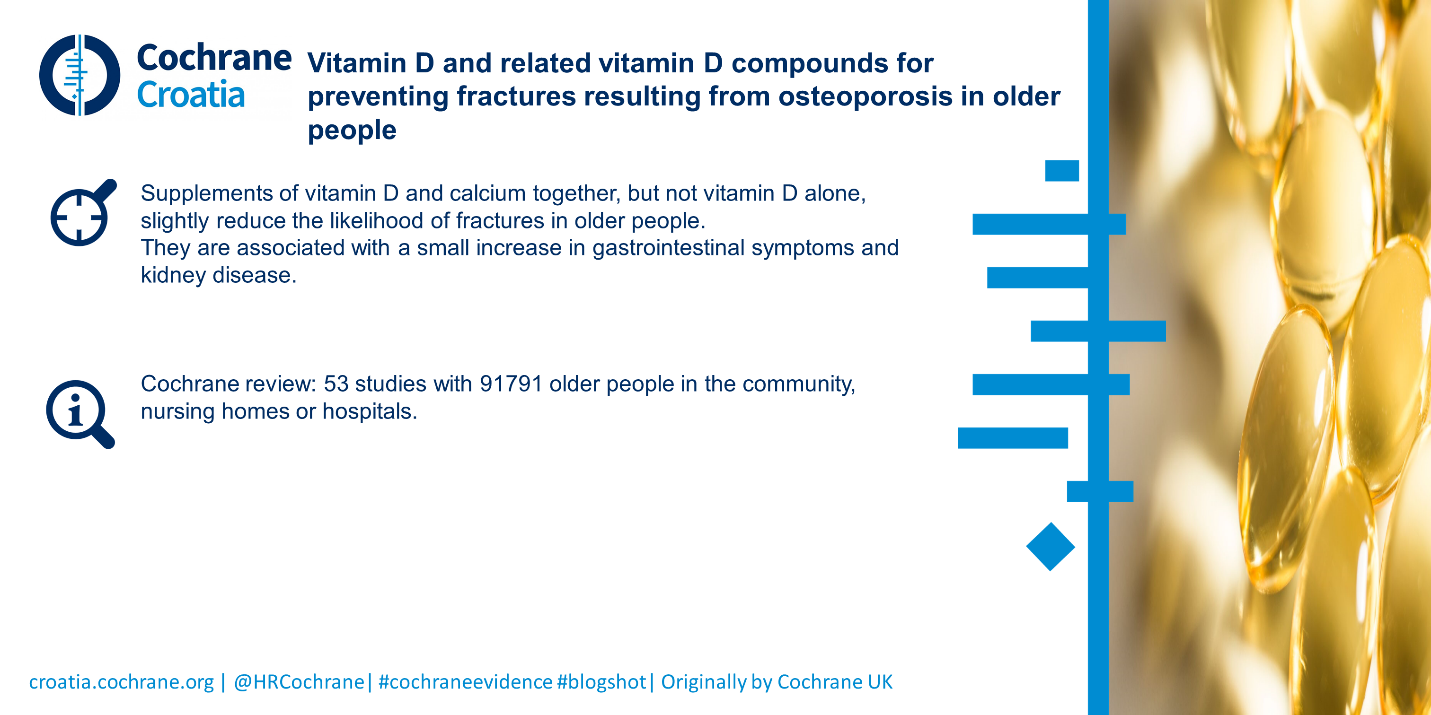


**Please answer the questions about the text**

1. For which medical condition the effectiveness of vitamin D was tested?

a) kidney disease b) indigestion c) fractures

2. Which compound, along with vitamin D, can help patients who suffer from described medical condition?

a) Placebo b) calcium c) It is not mentioned

3. How many trials were included in this review?

a) 91791 b) one c) 53

4. How many people with this health medical condition was included in this review?

a) 91791 b) one c) 53

**In the following three questions there are no correct or incorrect answers. We only ask your opinion about the present statements. Please choose the number which describes your opinion the most.**

**5. I consider vitamin D with calcium compounds is effective in prevention of fractures which are caused by osteoporosis in older people.**

| **I do not agree at all** |  |  |  |  |  |  |  |  | **I agree completely** |
| --- | --- | --- | --- | --- | --- | --- | --- | --- | --- |
| 1 | 2 | 3 | 4 | 5 | 6 | 7 | 8 | 9 | 10 |

**6. This is the appropriate format for health information presentation.**

| **I do not agree at all** |  |  |  |  |  |  |  |  | **I agree completely** |
| --- | --- | --- | --- | --- | --- | --- | --- | --- | --- |
| 1 | 2 | 3 | 4 | 5 | 6 | 7 | 8 | 9 | 10 |

**7. Please asses which way of presentation of health information do you prefer (-4 means that you prefer only words, and +4 means that you prefer only numbers)**

| **I prefer only words** |  |  |  |  |  |  |  | **I prefer only numbers** |
| --- | --- | --- | --- | --- | --- | --- | --- | --- |
| -4 | -3 | -2 | -1 | 0 | +1 | +2 | +3 | +4 |

**8. If you think that you would need another information to make a decision about the effectiveness of the vitamin D and its compounds, what would it be? Please state your opinion**

__________________________________________________________________________.

**In the following five questions there is only one correct answer. Please choose only one answer you think it is correct.**

1. A medical study will randomly assign people so they are equally likely to get medicine A or medicine B. If there are 300 people in the study, about how many are expected to get medicine A?

a. 100 people

b. 150 people

c. 200 people

d. 250 people

2. Older age and smoking both increase the risk of a heart attack over time. David is now 50 years old and smokes. His risk of a heart attack in the next 10 years is 10%. If he continues to smoke which of the following could be his risk of a heart attack over the next 20 years?

a. 5%

b. 10%

c. 30%

d. 100%

3. James starts a new blood pressure medicine. The chance of a serious side effect is 0.5%. If 1000 people take this medicine, about how many would be expected to have a serious side effect?

a. 1 person

b. 5 people

c. 50 people

d. 500 people

4. The PSA (Prostate Specific Antigen) is a blood test that can be used to screen for prostate cancer. However, 30% of men who have an abnormal test result will turn out not to have cancer. John has an abnormal test result. What is the chance that John has prostate cancer?

a. 0%

b. 30%

c. 70%

d. 100%

5. Rebecca is treated for stage 2 breast cancer. The chance that the cancer will come back is 10% over 10 years. If Rebecca takes a new medicine, this chance will decrease by 30%. If 100 women like Rebecca take this medicine, how many are now expected to have breast cancer come back within 10 years?

a. 3 out of 100 women

b. 7 out of 100 women

c. 10 out of 100 women

d. 30 out of 100 women

THE END!

Thank you for your participation!

**Appendix B**

**Qualitative study**

**Table 1.** Themes and quotations in focus groups discussions

| **Theme and characteristic** | **Quote** |
| --- | --- |
| **Influences on the choice of Characteristics health information source of information sources** | |
| The source of health information must be relevant and trusted by user. | *P8: (People who are my information source) …are the people I can trust to, people who do not hold anything back from me, and nothing remains unsaid...*  *M: How much do you think people believe the information found on Google?*  *P3: Very much...* |
| Widely spread information are over simplified. | *P5: (In the newspapers) All the conclusions are already there, and it says research has shown this and that…it is too simplified…* |
| The search process depends on two different factors: who is the patient and time limitation. | *P10: There we go back again to the context of the amount of time you have, because then you will not go searching through the literature systematically.*  *P11: It depends if it (information searching) is for you or for the baby. If it is for you, you will neglect it, but if it is for the baby, you will look for quality answers.* |
| **The Internet as the primary source of health information and other sources of information** | |
| Internet as the primary source for different populations. | *P1: Well, I think the Internet is the primary source, both for young and the old, while most books are now outdated…* |
| Most people do not search for specific websites but through forums. | *P13: I have found most of the things on forums, because those things you cannot find anywhere. You dig through a forum where there are 20 other people and they describe to you their experiences, therapy or whatever.* |
| Very few participants use specialized websites like Cochrane. | *M: How many of your patients would come with the information found on Cochrane websites?*  *P13: Nobody.*  *P15: I do not know if anyone has ever asked me about Cochrane.*  *P13: Sometimes they come with questions regarding scientific research, often found on pharmaceutical websites, but that is it…* |
| **Issues in comprehension of current scientific formats** | |
| Presentation of numbers is difficult to understand. | *P4: I think people avoid numbers.*  *P8: Here it says; “six babies per hundred”, and we do not know whether that is being summed, subtracted or what. We usually use percentages, and I do not know the way how people would have understood it better.* |
| Understanding of probability is difficult for lay population. | *P14: Let’s take risk for example. That is something that is hard to talk about because as soon as you are presenting numbers, whether it’s about mortality or effectiveness, that number can mean something, and it can mean nothing. For example, 40% mortality may be a good and a bad thing at the same time. And we can never be 100% certain about something in medicine.* |
| **Doctors and consumers have communication issues** | |
| The problem of communication is hardened because of high number of patients in general practice offices | *P12: We need more physicians, in order for everyone to have sufficiently big or low number of patients. The main problem is time, because if a general practitioner has 80 patients per day, it is impossible to improve communication. And general practitioners are the ones from which we expect best communication because they are the ones who know those patients.* |
| There is a feeling that doctors do not upgrade their knowledge sufficiently. | *P10: I have noticed that people who have graduated and started working immediately, after few years* *have issues with upgrading of new knowledge. They are not open to new experiences or anything new.* |
| **Recommendations for improvement** | |
| Current PLSs contain too much information which are not relevant to consumer, and that should be removed. | *P3: This “blinding” term is not necessary…That can be removed and write about the quality of the study…*  *M: So, according to you, methodology is irrelevant to the consumer.*  *P3: Yeah, that’s right.*  *M: And what would be relevant for them?*  *P2: To state what was the question and what are the results. Pure application in real world..* |
| Information about Cochrane should be in local general practices and available as one of the first choices online. | *P4: It would be good to have this type of information in general practices, or online on the first page on Google.* |
